# Post-Hoc Analysis on the Effects of Vicadrostat/Empagliflozin by CKD Severity/Hypertension Status

**DOI:** 10.1016/j.ekir.2026.106545

**Published:** 2026-04-16

**Authors:** Ricardo Correa-Rotter, Peter Rossing, Maria Eugenia Canziani, Maria Luiza Caramori, David Cherney, Lisa Cronin, Christian Hugo, Bo Ji, Juliane Meyerhoff, Masaomi Nangaku, Arnold Silva, Dorothea Urbach, Shimoli V. Shah, Dick de Zeeuw, Katherine R. Tuttle

**Affiliations:** 1 Instituto Nacional de Ciencias Médicas y Nutrición Salvador Zubirán, Mexico City, Mexico; 2 Steno Diabetes Center Copenhagen, Herlev, Denmark; 3 Department of Clinical Medicine, University of Copenhagen, Copenhagen, Denmark; 4 Universidade Federal de São Paulo, São Paulo, Brazil; 5 Cleveland Clinic Foundation, Cleveland, Ohio, USA; 6 University of Minnesota, Minneapolis, Minnesota, USA; 7 Division of Nephrology, Department of Medicine, University Health Network and University of Toronto, Toronto, Ontario, Canada; 8 Boehringer Ingelheim Pharmaceuticals, Ridgefield, Connecticut, USA; 9 Universitätsklinikum Carl Gustav Carus Dresden, Medizinische Klinik und Poliklinik III, Dresden, Germany; 10 Boehringer Ingelheim International, Ingelheim am Rhein, Germany; 11 University of Tokyo Hospital, Tokyo, Japan; 12 Boise Kidney and Hypertension, Meridian, Idaho, USA; 13 Clinical Trials Network Helderberg, Cape Town, South Africa; 14 Department of Clinical Pharmacy and Pharmacology, University Medical Centre Groningen, Groningen, Netherlands; 15 Providence Inland Northwest Health, Spokane, Washington, USA; 16 University of Washington, Seattle, Washington, USA

**Keywords:** albuminuria, aldosterone synthase inhibitor, chronic kidney disease, kidney function, risk factor

## Abstract

**Introduction:**

This phase 2 trial (NCT05182840) assessed the efficacy and safety of the aldosterone synthase inhibitor, vicadrostat, alone or with empagliflozin, in people with chronic kidney disease (CKD). These *post hoc* analyses assessed the effects of vicadrostat, with or without background empagliflozin, on albuminuria across a range of CKD severity and hypertension (HTN) status.

**Methods:**

The analysis set included 503 participants who had urine albumin-to-creatinine ratio (UACR) measures available at baseline and week 14. Subgroup analyses of the effects of vicadrostat, given alone or with empagliflozin, were performed by the presence or absence of uncontrolled HTN (defined as blood pressure [BP] ≥ 140/90 mm Hg and use of ≥2 classes of antihypertensive medication), kidney failure risk equation (KFRE) 4-factor 5-year risk < 5% versus ≥ 5%, Kidney Disease: Improving Global Outcomes (KDIGO) moderate-to-high risk versus very high risk, and diuretic use at baseline.

**Results:**

UACR was reduced with vicadrostat, with or without empagliflozin background, across all subgroup analyses (adjusted mean reduction with 10 mg and 20 mg vicadrostat dose groups: 25%–55% vs. 24%–51% in the overall study). UACR reductions were consistent in participants irrespective of KDIGO risk group, KFRE subgroup, presence or absence of uncontrolled HTN at baseline or diuretic use.

**Conclusion:**

The effects of vicadrostat on albuminuria reduction, with or without empagliflozin, were generally consistent irrespective of CKD stage and kidney risk as scored using KDIGO or the KFRE, HTN status, or use of diuretics.

## Graphical abstract

**Figure:**
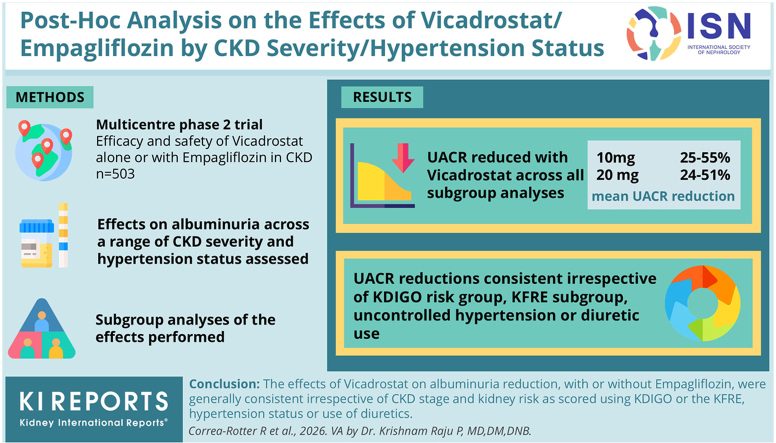

CKD remains a major clinical challenge despite the available treatments. Even with evidence-based interventions such as angiotensin-converting enzyme inhibitors, angiotensin receptor blockers, and sodium-glucose cotransporter 2 (SGLT2) inhibitors, people with CKD remain at high risk of continuous declining kidney function, cardiovascular (CV) events, and death.^1^, ^2^, ^3^, ^4^ The combined presence of cardio-kidney-metabolic (CKM) conditions, including type 2 diabetes (T2D), CKD, and CV disease, can amplify morbidity and mortality risks compared with the individual conditions.^5^, ^6^, ^7^

Aldosterone plays a central role in CKM conditions through mechanisms, including hemodynamic changes and direct cellular effects.^8^ Aldosterone promotes sodium retention and potassium excretion, which can contribute to HTN. In addition to hemodynamic effects, excess aldosterone may contribute to kidney damage through activation of the mineralocorticoid receptor,^9^ triggering inflammation and fibrosis.^10^ Elevated aldosterone levels are independently associated with an increased risk of CKD progression,^11^^,^^12^ with individuals who have high aldosterone levels facing a 45% greater risk of worsening kidney function than those with low aldosterone levels.^13^ Renin-angiotensin system inhibitors and nonsteroidal mineralocorticoid receptor antagonists can improve CKD outcomes but may not sufficiently block the deleterious effects of aldosterone bioactivity and can be associated with further increase of aldosterone levels.^14^ Unlike nonsteroidal mineralocorticoid receptor antagonists, which block aldosterone signaling at the receptor level, aldosterone synthase inhibitors suppress aldosterone production directly by inhibition of the CYP11B2 enzyme, which catalyzes the final steps in the synthesis of aldosterone.^10^ Given aldosterone’s central pathological role, direct inhibition of aldosterone synthase, the final enzyme in the biosynthesis of aldosterone, may provide additional therapeutic benefits to current standards of care with renin-angiotensin system inhibitors by reducing aldosterone production.

Vicadrostat is a potent and highly selective aldosterone synthase inhibitor that is being investigated in phase 3 trials when given with empagliflozin for the treatment of CKD (NCT06531824), heart failure (NCT06424288 and NCT06935370), and for the reduction of CV risk in patients with HTN, T2D, and established CV disease (NCT07064473). The efficacy and safety of vicadrostat, alone or with empagliflozin, was examined in a phase 2 multinational, randomized, dose-finding trial investigating people with CKD with or without T2D.^15^^,^^16^ The primary findings demonstrated UACR reductions of ≤40% with 10 mg or 20 mg vicadrostat, and ≤61% when given with empagliflozin.^15^ Participants receiving vicadrostat and empagliflozin together (VicaEmpa) showed UACR response rates (defined as a UACR reduction ≥ 30%) ≤ 70%.^15^ This degree of UACR reduction is a clinically relevant threshold that predicts long-term preservation of kidney function.^17^ We conducted *post hoc* analyses of the phase 2 trial data to assess the effects of vicadrostat, with or without background empagliflozin, on albuminuria across a range of CKD severity and HTN status.

## Methods

The phase 2 dose-finding trial design and methodology have been previously published (Supplementary Figure S1).^15^^,^^16^ Briefly, adults with CKD were eligible if they had an estimated glomerular filtration rate (eGFR) of 30 to < 90 ml/min per 1.73 m^2^ and UACR of 200 to < 5000 mg/g while receiving a maximally tolerated, stable dose of a renin-angiotensin system inhibitor. Participants were randomized (i.e., R1) 1:1 to receive background empagliflozin 10 mg or matching placebo (PBO_EMPA_) during an 8-week run-in. Participants were then rerandomized (i.e., R2) 1:1:1:1 to receive vicadrostat (3 mg, 10 mg, or 20 mg) or matching placebo (PBO_VICA_) for 14 weeks, in addition to their assigned empagliflozin or PBO_EMPA_. The primary end point was the change from R2 baseline in UACR at week 14 with UACR response (≥ 30% reduction from R2 baseline in UACR at week 14) as a secondary outcome. Additional outcomes included changes in systolic BP (SBP) and eGFR from R2 baseline to week 14. Adverse events (AEs) and safety data based on the treated set were assessed.

We performed subgroup analyses of the effects of vicadrostat, given alone or with background empagliflozin according to the following 4 categories: (i) by KDIGO moderate-to-high risk versus very high risk,^2^ (ii) by KFRE 4-factor 5-year risk (< 5% vs. ≥ 5%) based on UACR, eGFR, age, and sex (female vs. male),^18^ (iii) by the presence or absence of uncontrolled HTN (defined as SBP ≥ 140 mm Hg or diastolic BP ≥ 90 mm Hg and use of ≥ 2 classes of antihypertensive medication), and (iv) by diuretic use at baseline. SBP and diastolic BP were assessed to the nearest 1 mm Hg before blood sampling and performed in triplicate, with the mean of the 3 measurements used to confirm eligibility. Participants were stratified by the presence or absence of uncontrolled HTN at R1, and BP measurements were assessed at prespecified time points with participants in a seated position after 5 minutes of rest and recorded to the nearest 1 mm Hg.

### Statistical Methods

The primary efficacy analysis was based on the full analysis set (all participants randomly assigned at R2 baseline with ≥1 UACR measurements at week −2/−1 or 0 and ≥ 1 post-R2 measurement). Subgroup analyses of the primary outcome were performed for mixed models for repeated measures estimates of placebo-adjusted changes from R2 baseline in log-transformed UACR at week 14 for vicadrostat alone and with empagliflozin.^15^ Interaction terms between treatment and subgroup were included in the models to test for heterogeneity of treatment effects across subgroups. The same mixed models for repeated measures approach was applied to evaluate subgroup effects on change from baseline in eGFR and SBP. *P*_INTERACTION_ was calculated for each of the 4 subgroup analyses for treatment groups with and without empagliflozin. To account for multiple comparisons, *P*-values were adjusted using the Bonferroni correction method; the adjusted significance level was defined as 0.05/n where n represents the number of comparisons performed. All *P*-values presented are nominal. An adjusted multiple imputation method was used for missing UACR measurements at week 14 for the responder analysis. Descriptive statistics were used for the proportional UACR responses. Safety analyses were descriptive and included all treated participants.

## Results

### Study Population

As previously reported, of 714 participants initially randomized (R1), 586 continued to rerandomization (R2) forming the treated set (*n* = 583).^15^^,^^16^ UACR data were incomplete for 83 participants (full analysis set, *n* = 503). The overall mean age of participants was 63.8 years (SD: 11.3) and 33% (196/586) were female. Baseline demographic and clinical characteristics of the overall population were mostly similar across vicadrostat dose groups; selected parameters by subgroup are shown in Tables 1 and 2.

**Table 1:** Baseline characteristics of subgroups by KDIGO and KFRE risk at baseline

Characteristics	Overall	Moderate-to-high KDIGO risk class^a^	Very high KDIGO risk class	KFRE risk < 5%	KFRE risk ≥ 5%
	*N* = 586	*n* = 229 (39.1)	*n* = 345 (58.8)	*n* = 340 (58.0)	*n* = 246 (42.0)
Female, *n* (%)	196 (33.4)	79 (34.5)	111 (32.2)	124 (36.5)	72 (29.3)
Age, yrs (SD)	63.8 (11.3)	62.7 (11.0)	64.5 (11.4)	63.6 (10.5)	64.1 (12.3)
T2D status, *n* (%)	414 (70.6)	166 (72.5)	239 (69.3)	246 (72.4)	168 (68.3)
HbA1c, % (SD)	7.0 (1.3)	7.0 (1.2)	6.9 (1.4)	7.0 (1.3)	6.9 (1.4)
BMI, kg/m^2^ (SD)	29.9 (5.5)	30 (5.1)	29.9 (5.7)	30.1 (5.4)	29.7 (5.6)
Serum aldosterone, pmol/l, median (range)	125.5 (3–1984)	119.6 (3–821)	130.7 (3–1984)	125.8 (3–821)	125.1 (3–1984)
UACR, mg/g, (median IQR)	426 (205.3–889.5)	272.7 (165.8–606.5)	586.5 (312.7–1098.0)	330.3 (175.7–676.2)	693.1 (281.5–1287.3)
SBP, mm Hg (SD)	133.8 (15.7)	133.5 (13.7)	134.1 (16.9)	133.8 (15.2)	133.8 (16.4)
eGFR, ml/min per 1.73 m^2^ (SD)	51.9 (17.7)	67.8 (12.6)	40.6 (9.3)	63.2 (14.4)	36.5 (7.1)
NT-proBNP, ng/L (SD)	309.7 (625.4)	231.8 (562.1)	362.3 (667.6)	263.5 (634.6)	372.3 (608.5)

BMI, body mass index; eGFR, estimated glomerular filtration rate; HbA1c, glycated hemoglobin; IQR, interquartile range; KDIGO, Kidney Disease: Improving Global Outcomes; KFRE, kidney failure risk equation; NT-proBNP, N-terminal pro-B-type natriuretic peptide; SBP, systolic blood pressure; T2D, type 2 diabetes; UACR, urine albumin-to-creatinine ratio.

Missing baseline UACR data for 3 patients were not included in the subgroup analysis.

a The Moderate risk group count reflects the analytic definition used for this subgroup analysis, which did not include the eGFR G3a/UACR A1 criteria. An additional 9 patients meeting these specific criteria, also classified as moderate risk per KDIGO, were not included in this subgroup analysis but are accounted for in the overall study cohort.

**Table 2:** Baseline characteristics of subgroups by hypertension status and use of diuretics at baseline

Characteristics	Overall	Uncontrolled HTN	Controlled HTN	Diuretics, yes	Diuretics, no
	*N* = 586	*n* = 197 (33.6)	*n* = 389 (66.4)	*n* = 209 (35.7)	*n* = 377 (64.3)
Female, *n* (%)	196 (33.4)	56 (28.4)	140 (36.0)	65 (31.1)	131 (34.7)
Age, yrs (SD)	63.8 (11.3)	65.4 (9.4)	63.1 (12.0)	66.3 (8.7)	62.5 (12.3)
T2D status, *n* (%)	414 (70.6)	161 (81.7)	253 (65.0)	174 (83.3)	240 (63.7)
HbA1c, % (SD)	7.0 (1.3)	7.2 (1.3)	6.8 (1.3)	7.4 (1.3)	6.7 (1.3)
BMI, kg/m^2^ (SD)	29.9 (5.5)	30.6 (5.2)	29.6 (5.6)	31.3 (5.7)	29.1 (5.2)
Serum aldosterone, pmol/l, median (range)	125.5 (3–1984)	132.1 (3–821)	124.3 (3–1984)	131.2 (3–781)	123.2 (3–1984)
UACR, mg/g, (median, IQR)	426 (205.3–889.5)	558.7 (246.5–1059.7)	380.5 (187.3–828.3)	422.5 (187.5–872.2)	432.3 (222.3–902.0)
SBP, mm Hg (SD)	133.8 (15.7)	142.7 (15.0)	129.2 (14.0)	135.0 (16.2)	133.1 (15.4)
eGFR ml/min per 1.73 m^2^ (SD)	51.9 (16, 17.7)	50.7 (17.0)	52.6 (18.1)	50.8 (16.5)	52.6 (18.4)
NT-proBNP, ng/L (SD)	309.7 (625.4)	367.1 (580.4)	280.4 (645.9)	320.4 (501.7)	303.4 (607.5)

BMI, body mass index; eGFR, estimated glomerular filtration rate; HbA1c, glycated hemoglobin; HTN, hypertension; IQR, interquartile range; NT-proBNP, N-terminal pro-B-type natriuretic peptide; SBP, systolic blood pressure; T2D, type 2 diabetes; UACR, urine albumin-to-creatinine ratio.

Missing baseline UACR data for 3 patients were not included in the subgroup analysis.

Of the 583 participants in the present analyses, 229 participants (40%) were categorized as having moderate-to-high KDIGO risk and 345 (60%) had very high risk. The moderate-to-high risk group count reflects the analytic definition used for this analysis and did not include patients with eGFR G3a/UACR A1. Therefore, 9 patients meeting these specific criteria, who would also be classified as moderate risk per KDIGO, were not included in this subgroup analysis. Baseline demographic and clinical characteristics were generally similar between KDIGO risk class groups except for higher serum aldosterone (median: 130.7 vs. 119.6 pmol/l), lower eGFR (mean 40.6 vs. 67.8 ml/min per 1.73 m^2^), N-terminal pro-B-type natriuretic peptide (362.3 vs. 231.8 ng/l), and higher UACR (mean: 586.5 vs. 272.7 mg/g) in the very high risk group versus the moderate-to-high risk group. Two-hundred forty-six trial participants (42%) had a KFRE risk ≥ 5% at baseline; these participants had similar baseline characteristics to the KFRE < 5% risk group except for a higher UACR (mean: 693.1 vs. 330.3 mg/g) and lower eGFR (36.5 vs. 63.2 ml/min per 1.73 m^2^), these being 2 of the parameters used in the KFRE, and N-terminal pro-B-type natriuretic peptide (372.3 vs. 263.5 ng/l, respectively). Uncontrolled HTN was present in 197 (trial participants 33.6%) at baseline; in addition to a higher SBP (mean: 142.7 vs. 129.2 mm Hg), these participants were more likely to have a prior diagnosis of T2D (81.7% vs. 39.3%), higher N-terminal pro-B-type natriuretic peptide (367.1 vs. 280.4 ng/l), and a higher average UACR (558.7 vs. 380.5 mg/g) at baseline. Of the trial participants, 63 (11%) had treatment-resistant HTN, defined as having a BP measurement ≥ 140/90 mm Hg at baseline with ≥3 antihypertensive medications. At baseline, 209 trial participants (35.7%) were receiving diuretics, a higher proportion of whom had a previous diagnosis of T2D (83.3% vs. 63.7%).

### Change in UACR to Week 14

**Figure 1:**
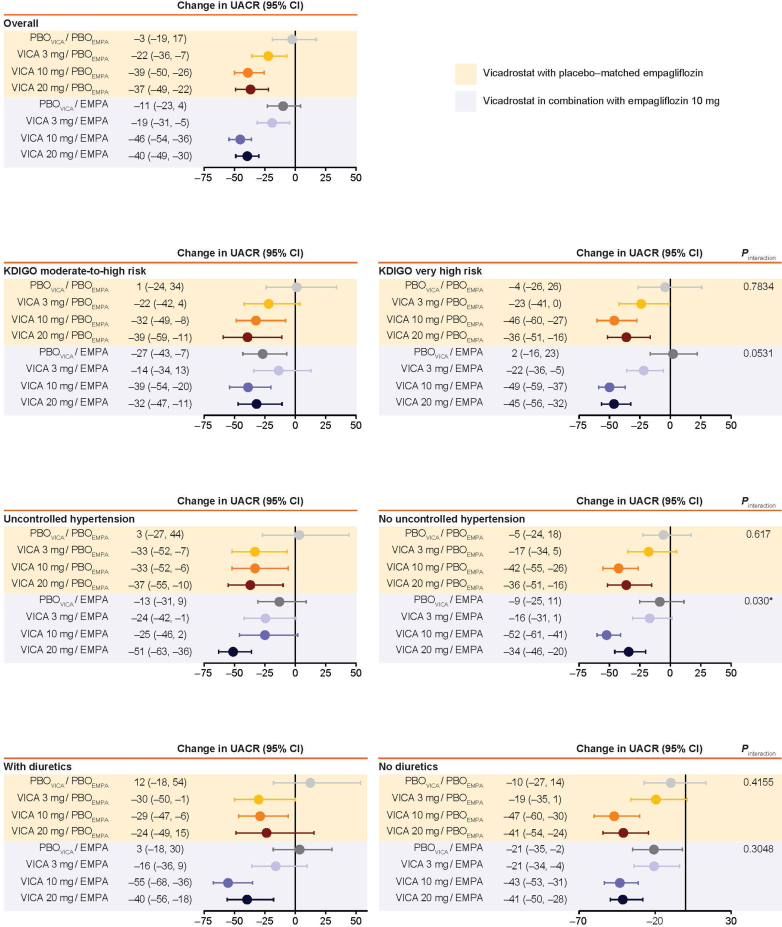
Adjusted geometric mean change (95% CI) in log-transformed UACR from baseline to end of treatment at week 14. ∗Not significant after Bonferroni correction for multiple analyses. CI, confidence interval; EMPA, empagliflozin; PBO, placebo; UACR, urine albumin-to-creatinine ratio; VICA, vicadrostat.

Vicadrostat, with or without empagliflozin, reduced UACR across all subgroup analyses (adjusted mean reduction with 10 mg and 20 mg vicadrostat dose groups: 24%–55% vs. 37%–46% in the overall study) as shown in Figure 1 (KFRE data in Supplementary Figure S2A).

Consistent reductions in placebo-adjusted percentage UACR change from baseline was observed across KDIGO risk groups at all vicadrostat doses tested (P_INTERACTION_ = 0.053 and 0.783, with and without empagliflozin, respectively); consistent reductions in UACR were observed across KFRE subgroups (P_INTERACTION_ = 0.238 and 0.910). Reductions in UACR with vicadrostat versus PBO_VICA_ were observed in participants with and without uncontrolled HTN at baseline (P_INTERACTION_ = 0.030 and 0.617; P_INTERACTION_ with empagliflozin was not significant after correcting for multiple comparisons). Furthermore, the direction of the dose response with empagliflozin was consistent but with an apparent difference in intensity between HTN subgroups. Vicadrostat consistently reduced UACR in participants irrespective of diuretic use at baseline (P_INTERACTION_ = 0.305 and 0.416, with and without empagliflozin, respectively).

### UACR Responder Analysis (≥ 30% UACR Reduction From Baseline to Week 14)

**Figure 2:**
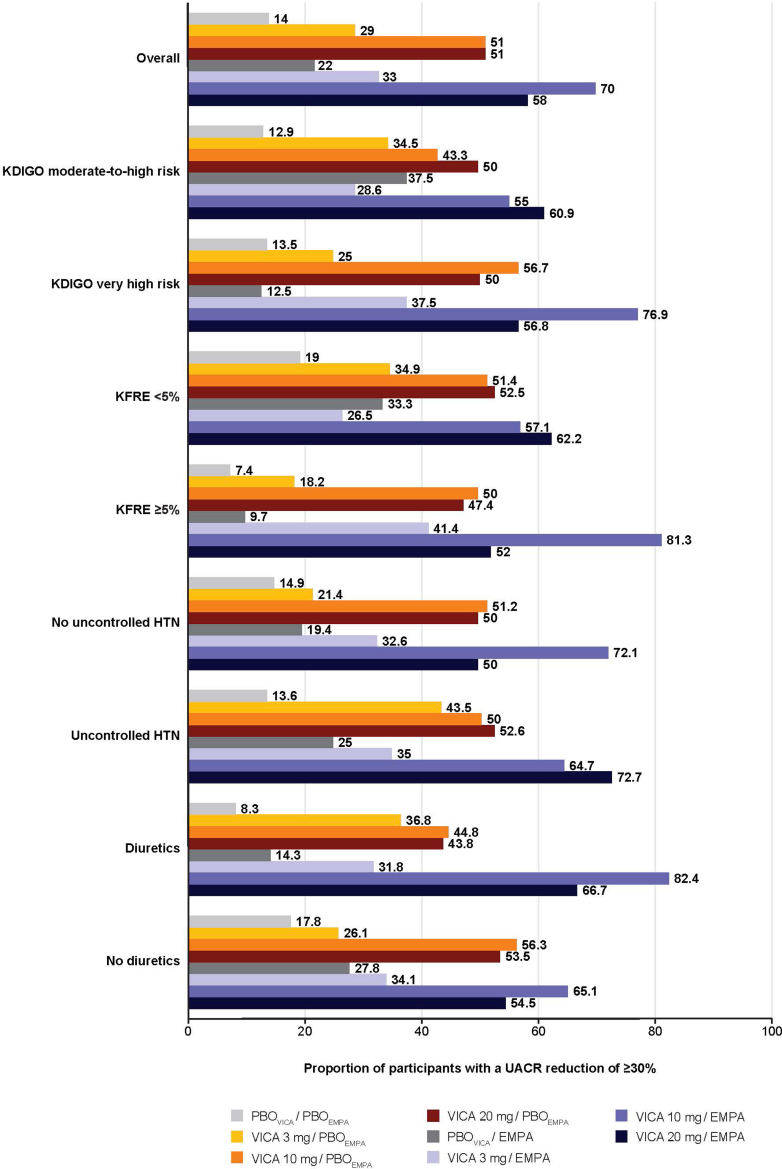
Proportion of participants with a UACR reduction of ≥ 30% from baseline to end of treatment at week 14. EMPA, empagliflozin; KDIGO, Kidney Disease: Improving Global Outcomes; KFRE, kidney failure risk equation; PBO, placebo; UACR, urine albumin-to-creatinine ratio; VICA, vicadrostat.

In this analysis, the UACR response rate across all kidney risk subgroups showed improvement that was consistent with that observed in the overall population, with a greater proportion of participants meeting a ≥30% UACR reduction from baseline to week 14 in the 10 mg and 20 mg groups. A UACR response (≥ 30% reduction) was achieved in 43% to 82% of participants in the vicadrostat 10 mg and 20 mg dose groups across all subgroups, with a numerically greater number of responders observed with VicaEmpa versus vicadrostat alone (Figure 2). Response rates with VicaEmpa were higher in the treatment groups who were taking diuretics at baseline (82% in the 10 mg and 67% in the 20 mg vicadrostat dose group, respectively) than in those who were not taking diuretics (65% and 55%, respectively). In contrast, UACR response rates with vicadrostat 10 mg and 20 mg doses without background empagliflozin tended to be higher in groups who were not on diuretics at baseline (56% and 54% in the 10 mg and 20 mg vicadrostat dose groups without diuretics vs. 45% and 44%, respectively, with diuretics).

### SBP

**Figure 3:**
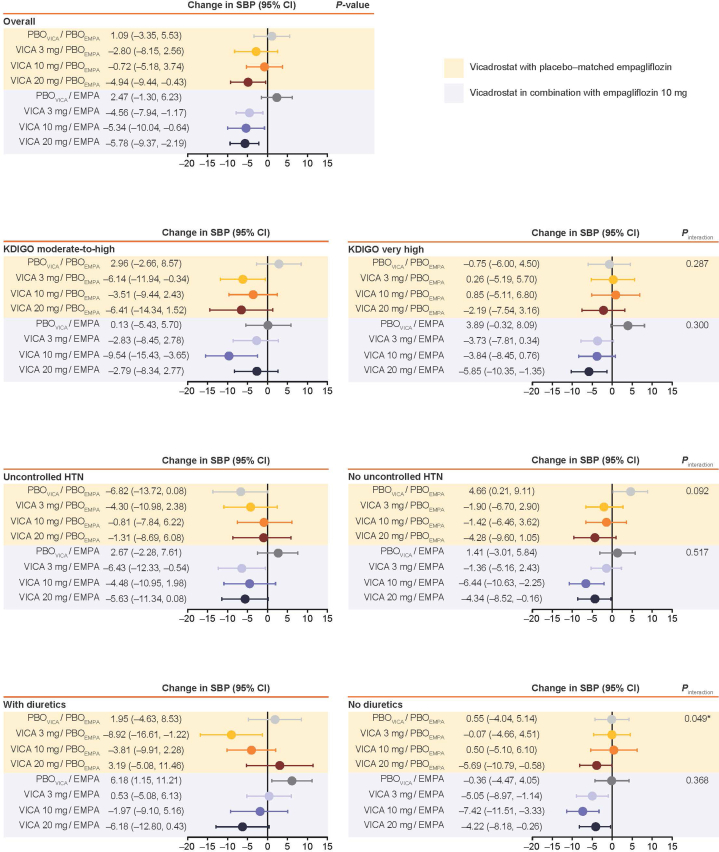
Change in SBP from baseline to week 14, mm Hg (95% CI). ∗Not significant after Bonferroni correction for multiple analyses. CI, confidence interval; EMPA, empagliflozin; HTN, hypertension; KDIGO, Kidney Disease: Improving Global Outcomes; PBO, placebo; SBP, systolic blood pressure; VICA, vicadrostat.

Vicadrostat was associated with consistent reductions in SBP that were comparable across all subgroups, including participants who had uncontrolled HTN at baseline (Figure 3, KFRE data in Supplementary Figure S2B). SBP reductions were generally greater in participants who received background empagliflozin across all subgroups.

Reductions in SBP from baseline were observed in both KDIGO risk groups, with no apparent trends in SBP change among dose groups or between the moderate-to-high versus very high-risk classes (P_INTERACTION_ = 0.300 and 0.287, with and without empagliflozin, respectively). SBP change was consistent between KFRE risk groups (P_INTERACTION_ = 0.429 and 0.490). Changes in SBP across the tested doses of vicadrostat tended to be greater with versus without empagliflozin background, irrespective of HTN status at baseline (P_INTERACTION_ = 0.517 and 0.092), with no clear dose dependency observed across the categories. Reductions in SBP were observed with vicadrostat irrespective of the use of diuretics at baseline (P_INTERACTION_ = 0.368 and 0.049; P_INTERACTION_ without empagliflozin was not significant after correcting for multiple comparisons).

### eGFR

**Figure 4:**
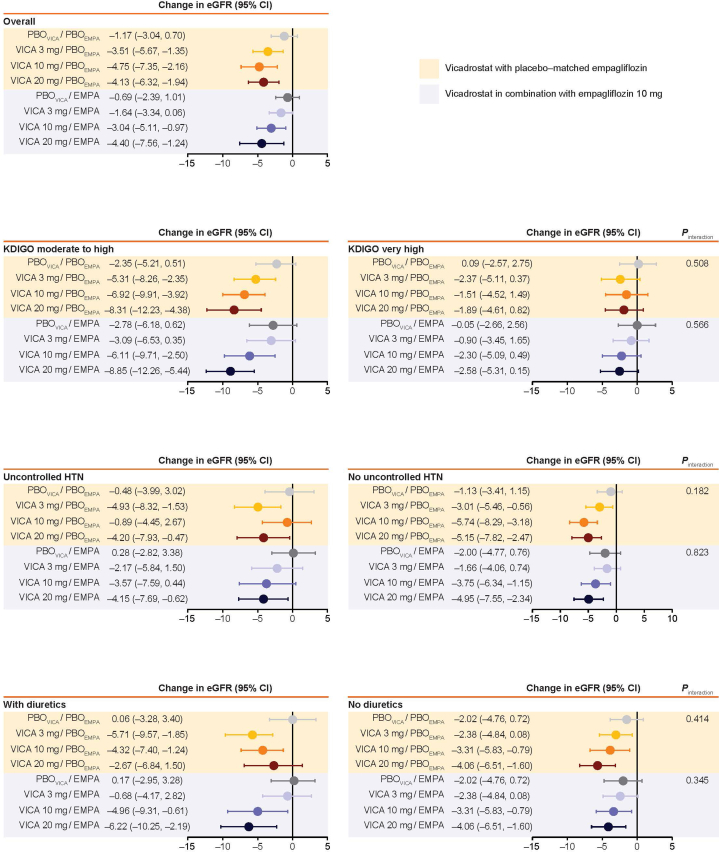
Change in eGFR from baseline to week 14 (ml/min per 1.73 m^2^). CI, confidence interval; eGFR, estimated glomerular filtration rate; EMPA, empagliflozin; HTN, hypertension; KDIGO, Kidney Disease: Improving Global Outcomes; PBO, placebo; VICA, vicadrostat.

Overall, modest changes in eGFR from baseline to week 14 ranging from −1.64 to −4.75 ml/min per 1.73 m^2^ occurred in the vicadrostat treatment groups, ranging from −0.17 to −8.85 across the subgroups tested (Figure 4, KFRE data in Supplementary Figure S2C).

Changes in eGFR from baseline were consistent in both KDIGO risk groups (P_INTERACTION_ = 0.566 and 0.508, with and without empagliflozin, respectively) and in both KFRE risk groups (P_INTERACTION_ = 0.401 and 0.782, with and without empagliflozin, respectively). Compared with participants in the moderate-to-high KDIGO risk group, participants in the very high KDIGO risk group did not tend to have a substantial eGFR decline, and a similar pattern was observed with participants in the KFRE ≥ 5% group compared with participants in KFRE < 5% subgroup. In HTN subgroups, changes in eGFR appeared to be dependent on vicadrostat dose in those with empagliflozin background, and the magnitude of eGFR changes was comparable in those with controlled versus uncontrolled HTN at baseline (P_INTERACTION_ = 0.823 and 0.182). Changes in eGFR were consistent in participants irrespective of diuretic use (P_INTERACTION_ = 0.414 and 0.345).

### Safety

The overall safety for vicadrostat with and without empagliflozin has been previously reported.^15^ A summary of AEs, serious AEs, and AEs leading to discontinuation of vicadrostat for all subgroups is shown in Supplementary Table S1.

The frequency of AEs (all types) was similar between KDIGO risk class groups, KFRE risk groups, and irrespective of background diuretic use, with no clear patterns by vicadrostat dose or use of background empagliflozin. The frequency of any AE was comparable in participants with controlled versus uncontrolled HTN, irrespective of background empagliflozin use.

Investigator-reported hypotension occurred in only 1.4% of trial participants (*n* = 8 [VicaEmpa 10/10 mg, *n* = 4; VicaEmpa 20/10 mg, *n* = 2; PBO_VICA_/empagliflozin, *n* = 1; vicadrostat 3 mg/PBO_EMPA_, *n* = 1]) as previously reported,^15^ precluding comparisons by vicadrostat dose or empagliflozin treatment.

## Discussion

This *post hoc* analysis of a phase 2, double-blind, placebo-controlled study demonstrates that vicadrostat was associated with clinically meaningful reductions in UACR in adults with CKD across a broad range of CKD stages, in the presence or absence of uncontrolled HTN or concomitant use of diuretics at baseline. Notably, responder rates (≥ 30% UACR decrease) were numerically greater for vicadrostat 10 mg or 20 mg combined with empagliflozin than in those treated with vicadrostat alone across all subgroups assessed.

High SBP increases the risk for CV events and mortality as well as CKD progression to kidney failure.^19^^,^^20^ Vicadrostat decreased SBP in people with CKD, with reductions that were comparable across all subgroups in this analysis, including participants who had uncontrolled HTN at baseline. These reductions were generally greater in participants who received background empagliflozin, consistent with the trend observed in the overall population. Furthermore, the data reported here are for the vicadrostat treatment period (with R2 as baseline) and are in addition to the reductions observed during the run-in period where reductions of −2.2 mm Hg were observed in patients receiving empagliflozin compared with those who received matched placebo.^21^

Regarding kidney function changes, the observed eGFR changes were generally modest and consistent with the expected glomerular hemodynamic effects of vicadrostat and empagliflozin. Notably, participants in the very high KDIGO risk group and KFRE > 5% group tended not to have a substantial eGFR decrease in absolute terms, possibly because of the lower eGFR of these groups at baseline. Hemodynamic effects in the kidney were expected, based on the mechanism of action of both vicadrostat and empagliflozin. The observed eGFR changes were generally modest. Notably, an initial eGFR decline (also known as eGFR dip) of approximately 3 to 5 ml/min per 1.73 m^2^ following initiation of treatment has been reported across SGLT2 inhibitor trials,^22^, ^23^, ^24^, ^25^, ^26^ and a similar initial, reversible eGFR decline has been reported with renin-angiotensin-aldosterone inhibitors.^27^ However, a meta-analysis with individual patient-level data showed that SGLT2 inhibition reduced risk of kidney outcomes irrespective of the size of the acute dip in eGFR.^28^

Diuretics are frequently used in patients with CKD or heart failure to manage volume overload or to reduce BP and are likely to be common in this patient population, who would likely benefit from vicadrostat use. Empagliflozin has previously shown consistent effects in patients regardless of diuretic use,^29^ and the present analysis indicates that vicadrostat maintained efficacy with and without concomitant diuretic therapy. Although some variation in responder rates by background empagliflozin use was observed, all were clinically meaningful and ≥40% at the vicadrostat 10 mg and 20 mg doses.

Separate subanalyses have shown that the effects of VicaEmpa or vicadrostat monotherapy on UACR, eGFR, and SBP were consistent, irrespective of T2D and obesity status (Cherney DZI, *et al.* Diabetes Obesity and Metabolism: IN PRESS). Taken together with the current analyses, these findings indicate a reliable effect of vicadrostat and VicaEmpa across a broad range of patients with CKD, with no unexpected safety findings identified in the subgroups analyzed. This broad patient population will be reflected in the ongoing phase 3 EASi-KIDNEY trial (NCT06531824) that compares VicaEmpa versus empagliflozin, and includes patients with and without albuminuria, with and without T2D, and across a broad range of kidney function (down to an eGFR of 20 mg/ml per 1.73 m^2^). This contrasts with many other studies in CKM conditions, such as those for baxdrostat/dapagliflozin or finerenone, where inclusion criteria were more restrictive, limited to patients who had both CKD and HTN such as BaxDuo-ARCTIC,^30^ or CKD and T2D, including FIDELIO-DKD, FIGARO, CONFIDENCE, and FLOW.^31^, ^32^, ^33^, ^34^

Considering that UACR is a known marker of CV risk as well as kidney risk, the results of this analysis support the inclusion of a broad range of patients in a wider VicaEmpa phase 3 clinical trial program. Therefore, in addition to the EASi-KIDNEY trial, VicaEmpa is being evaluated compared with standard of care including empagliflozin for the treatment of heart failure (EASi-HF Preserved, NCT06424288; EASi-HF Reduced, NCT06935370) and to reduce CV risk in people with T2D, HTN, and established CV disease (EASi-PROTKT, NCT07064473). The growing interest in combination therapies for CKD is also reflected by the CONFIDENCE trial, which demonstrated that inhibition of both the mineralocorticoid receptor (finerenone) and SGLT2 (empagliflozin) yields additive reductions in albuminuria compared with either agent alone.^31^ This supports the concept that targeting multiple pathogenic pathways may enhance efficacy in CKD, reflected by augmented UACR lowering, and highlights the clinical relevance of evaluating novel combinations including aldosterone synthase inhibitors with SGLT2 inhibitors.

This analysis has several limitations. First, the phase 2 trial was not powered for these *post hoc* subgroup analyses. Second, several of the different subgroups assessed are closely associated, particularly KDIGO risk and KFRE; all 229 in the moderate-to-high KDIGO risk group had a KFRE risk < 5%, whereas the very high KDIGO risk group included all 244 of the KFRE ≥ 5% group and 101 participants from the KFRE < 5% group. This interdependence should be considered when interpreting these results. Although there is currently much attention on aldosterone synthase inhibition as a potential treatment for HTN, this phase 2 trial was not designed to assess BP, and mean baseline SBP was only modestly increased. The observed changes in SBP were therefore smaller than in other dedicated trials for aldosterone synthase inhibitor in uncontrolled or resistant HTN, such as BaxHTN and Launch-HTN, and these results should not be directly compared.^35^^,^^36^ Although none of the subgroup interaction terms remained significant after adjustment for multiple comparisons, raising the possibility of a type II error, the consistency in treatment effect estimates across all subgroups argues against clinically meaningful heterogeneity. The study was not powered to detect subgroup-specific interactions; given the exploratory nature and number of subgroup comparisons, findings should be interpreted as descriptive rather than confirmatory. Finally, the sample size in this analysis precluded definitive assessment of the link between aldosterone levels and outcomes, though this is of interest and may be possible in upcoming phase 3 studies that will include a greater number of participants.

The effects of vicadrostat on albuminuria reduction, whether given alone or with empagliflozin, were generally consistent irrespective of CKD stage and kidney risk as scored by KDIGO or KFRE, uncontrolled HTN status, or use of diuretics at baseline. Vicadrostat meaningfully decreased SBP in people with CKD, with reductions that were comparable across all subgroups analyzed, including participants with uncontrolled HTN, whereas eGFR changes were generally modest and consistent with expected hydrodynamic effects.

## Disclosure

RCR reported participation as a trial investigator for AstraZeneca, Chinook, Lilly, and Novo Nordisk; consulting fees from AstraZeneca, Bayer, Boehringer Ingelheim, Chinook, Lilly, and Novo Nordisk; payment of honoraria for lectures from Amgen, AstraZeneca, Bayer, Boehringer Ingelheim, and Novo Nordisk; and voluntary membership of the Diabetes Committee of SLANH and the Steering Committee for World Kidney Day. PR reported grants and payment of honoraria for lectures, educational events, and steering group participation from AstraZeneca, Bayer, and Novo Nordisk (all to the Steno Diabetes Center Copenhagen); payment of honoraria for lectures and participation in advisory boards from Abbott, Astellas, Boehringer Ingelheim, and Sanofi (all to the Steno Diabetes Center Copenhagen). MEC reported grants for investigator-initiated research and research funding from Baxter and Fresenius; and payment of honoraria for lectures, presentations, and education events from AstraZeneca, Bayer, Bracepharma, Fresenius, and Pfizer. MLC reported research grant support from Bayer Pharmaceuticals, Boehringer Ingelheim, Breakthrough T1D, Eli Lilly, NIDDK, and NIH (all to the Cleveland Clinic Foundation); consulting fees from Armana, AstraZeneca, Bayer Pharmaceuticals, Novo Nordisk, and Roche; payment for speaker bureaus and educational events from Bayer Pharmaceuticals; payment of honoraria for lectures and educational events from the American College of Cardiology, Cardiorenal Connections, Heart in Diabetes, and Translational Medicine Academy; support to attend investigator meetings for Kidney Precision Medicine Project from NIDDK and NIH, American Diabetes Association meetings from NIDDK and NIH and Cleveland Clinic Foundation, and American Society of Nephrology meetings from NIDDK and NIH and Cleveland Clinic Foundation; participation on and site principal investigator for the Data Safety Monitoring Board for Preventing Early Renal Loss in Diabetes Study for NIDDK and NIH (all to the University of Minnesota); and attendee of Kidney Disease Improving Global Outcomes writing group meetings. DZIC has received honoraria from AbbVie, Altimmune, Amgen, AstraZeneca, Bayer, Biobridge, Boehringer Ingelheim-Lilly, Bristol Myers Squibb, CSL-Behring, Gilead, GSK, Inversago, Janssen, Lexicon, Maze, Merck, Mitsubishi Tanabe, Novartis, Novo Nordisk, Otsuka, Prometic, Sanofi, Vantage, and Youngene; and has received operational funding for clinical trials from AstraZeneca, Bayer, Boehringer Ingelheim-Lilly, CSL-Behring, Janssen, Lexicon, Merck, Novo Nordisk, and Sanofi. MN reported donations for research through Shogaku Kifu practice from Boehringer Ingelheim, Chugai, Daiichi Sankyo, JT Pharma, Kyowa Kirin, Mitsubishi Tanabe, Takeda, and Torii; consulting fees from Kyowa Kirin and Mitsubishi Tanabe; and payment of honoraria for lectures from Astellas, AstraZeneca, Bayer, JT Pharma, Kyowa Kirin, and Mitsubishi Tanabe. AS reported research contracts from Boehringer Ingelheim, Mineralys, Novartis, ProKidney, and Reata; consulting fees from Ardelyx, Boehringer Ingelheim, and ProKidney; payment of honoraria for presentations from AstraZeneca, Bayer, Boehringer Ingelheim, and ProKidney; and participation on an Advisory Board for Boehringer Ingelheim, Reata, and Travere.

DdZ reported consulting fees from Bayer, Fresenius, and Travere. KRT reported grants for investigator-initiated research from Bayer, Goldfinch Bio, NCATS, NHLBI, NIDDK, NIH Data Science office, NIMHD, and Travere; contracts from CDC; consulting fees from AstraZeneca, Bayer, Boehringer Ingelheim, Eli Lilly, Gilead, Janssen, Merck Sharp & Dohme, and Novo Nordisk; payment for manuscript writing for Bayer, Boehringer Ingelheim, Eli Lilly, Gilead, and Novo Nordisk; payment of honoraria for AstraZeneca, Bayer, Eli Lilly, Gilead, and Novo Nordisk; payment for travel for Novo Nordisk; travel to meetings from Novo Nordisk; chair and member of a data safety monitoring committee for George Clinical and NIDDK; and leadership role for the American Society of Nephrology. BJ, JM, LC, and SVS are employees of Boehringer Ingelheim. All the other authors declared no competing interests.

## Supplementary Material

Supplementary File (PDF) **Figure S1.** NCT05182840 phase 2 trial study design. **Figure S2.** KFRE subanalyses for (A) UACR change from baseline, (B) SBP change from baseline, and (C) eGFR change from baseline. **Table S1.** Summary of investigator-reported adverse events during the treatment period. Consort checklist.
